# Functional characterization of the complement receptor type 1 and its circulating ligands in patients with schizophrenia

**DOI:** 10.1186/1472-6890-11-10

**Published:** 2011-08-25

**Authors:** Arsen Arakelyan, Roksana Zakharyan, Aren Khoyetsyan, David Poghosyan, Rouben Aroutiounian, Frantisek Mrazek, Martin Petrek, Anna Boyajyan

**Affiliations:** 1 Institute of Molecular Biology, National Academy of Sciences of the Republic of Armenia, 7 Hasratyan St., 0014, Yerevan, Armenia; 2 Faculty of Medicine and Dentistry, Palacky University, 6 I. P. Pavlova St., 775 20, Olomouc, Czech Republic; 3 Biological Faculty of Yerevan State University, 1 Al. Manoogian St., 0025, Yerevan, Armenia

## Abstract

**Background:**

Whereas the complement system alterations contribute to schizophrenia, complement receptors and regulators are little studied. We investigated complement receptor type 1 (CR1) expression on blood cells, the levels of circulating immune complexes (CIC) containing ligands of CR1, C1q complement protein and fragments of C3 complement protein (C1q-CIC, C3d-CIC), and CR1 C5507G functional polymorphism in schizophrenia patients and controls.

**Results:**

We found an increased C1q-CIC level and CR1 expression on blood cells, elevated number of CR1 positive erythrocytes and reduced number of CR1 positive lymphocytes and monocytes in patients compared to controls. No difference in the levels of C3d-CIC between groups was observed. Higher CR1 expression on erythrocytes in CC genotype versus CG+GG for both groups was detected, whereas no difference was observed for other cell populations. Our results indicated that schizophrenia is associated with the increased CR1 expression and C1q-CIC level.

**Conclusions:**

Our study for the first time indicated that schizophrenia is associated with the increased CR1 expression and C1q-CIC level. Further studies in other ethnic groups are needed to replicate these findings.

## Background

Complement receptor type 1 (CR1; CD35; C3b/C4b receptor) is a multifunctional receptor, which is expressed in the majority of peripheral blood cells [1-4], with high affinity to complement components C1q, C3, C4 [5-8] and mannose-binding lectin [9]. Binding of CR1 to opsonic fragments of the complement C3 and C4 components (C3b, C4b, iC3b, and C3dg) and to the complement component C1q attached to immune complexes (IC), foreign or damaged host cells serves to mediate clearance of IC (on erythrocytes (E)) [4,10] and phagocytosis of complement-coated particles (on leukocytes) [3,11,12]. In addition, interaction of CR1 with its ligands plays further roles in regulation of lymphocyte (L) activity by promoting secretion of interleukin (IL)-1α, IL-1β, and prostaglandins [13]. Furthermore, CR1 plays a role in antigen presentation to B cells [12]. In addition to membrane-bound CR1, leukocytes also release soluble form of CR1, which is a potent inhibitor of both the classical and the alternative pathways of complement activation by exhibiting decay-accelerating activity for both C3 and C5 convertases, as well as cofactor activity for factor I-mediated cleavage of C3b and C4b [10,13,14].

A number of studies suggest the involvement of alterations in the immune response, including autoimmune and inflammatory mechanisms, in the aetiopathogenesis of schizophrenia [15-17]. Alterations in functional activities of classical, alternative and lectin pathways of complement, a major mediator of the immune response, as well as changes in blood levels and expression profiles of its C1q, C3, and C4 components have been detected in schizophrenia-affected subjects [18-22]. However, little is known about receptors and regulators of the complement system in schizophrenia. Here CR1 represents a special interest, accounting for a positive genome-wide linkage of schizophrenia with the *CR1 *gene encoding locus (1q32) [23]. A common *CR1 *C5507G single nucleotide polymorphism (SNP) in exon 33 has been shown to be associated with some diseases characterized by altered inflammatory response [24,25], and changes in CR1 expression have been shown in patients with a variety of inflammatory and autoimmune diseases [14,26-28].

In the present study we investigated the expression of CR1 on E and leukocytes in patients with schizophrenia and evaluated the possible association of *CR1 *C5507G functional polymorphism with this disorder. In addition, in the blood of schizophrenia affected subjects the levels of circulating immune complexes (CIC) containing natural ligands of CR1, namely C1q and fragments of C3, were also determined. A control group included healthy subjects without family history of schizophrenia.

## Results

### CR1 expression on blood cells

**Figure 1:**
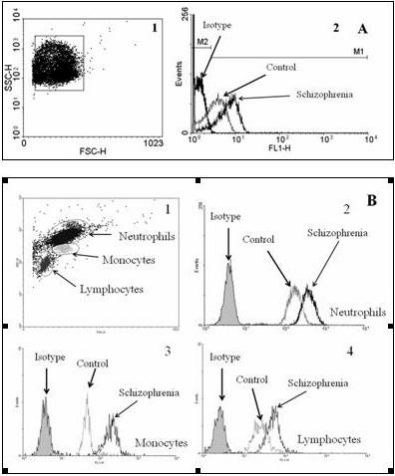
**Representative histograms of CR1 measurement on blood cells**. (A) CR1 measurement on erythrocytes: 1 - gated erythrocyte population. 2 - three fluorescent peaks representing non-specific (an isotype control) and specific fluorescence (schizophrenic patients and control subjects). (B) CR1 measurement on leukocytes: 1 - gated leukocyte populations. 2, 3, 4 - CR1 expression on neutrophils, monocytes, and lymphocytes, respectively.

The expression of CR1 on blood cells was quantified by flow cytometry. The results obtained are shown on Figure 1 and in table 1. According to the data obtained, CR1 expression levels for erythrocytes were significantly higher in patients compared to controls and were positively correlated with the duration of schizophrenia (rho = 0.637, *p *= 0.004). The same applies to the percentage of CR1-positive E (patients: 83.84 [2.66] % versus controls: 75.94 [6.74] %, *p *= 7.48E-07). Interestingly, the CR1 expression on subpopulation of leukocytes (L, monocytes (M), and neutrophils (N)) in patients was significantly higher than in controls (table 1), whereas the percentage of CR1 positive L (patients: 11.69 [4.03] % versus controls: 17.26 [6.60] %, *p *= 4.02E-05) and M (patients: 1.49 [0.91] % versus controls: 5.19 [1.83] %, *p *= 1.42E-10) was significantly lower. No significant difference was observed in the number of positive N among studied groups (patients: 64.18 [12.15] % versus controls: 59.93 [9.60] %, *p *= 0.52). Also, there was no difference in CR1 expression between smokers and non-smokers in both groups.

**Table 1:** CR1 expression on blood cells in schizophrenic patients and controls.

Type of blood cells	CR1 expression, RFI		*p*
	**Patients**	**Controls**	

Erythrocytes	6.85 [0.89]	6.21 [0.67]	0.001

Lymphocytes	22.35 [8.35]	19.30 [7.90]	0.011

Monocytes	85.55 [46.35]	47.30 [10.00]	1.20E-08

Neutrophils	185.5 [80.00]	140.00 [44.5]	0.003

### The influence of CR1 C5507G polymorphism on CR1 expression on blood cells

**Figure 2:**
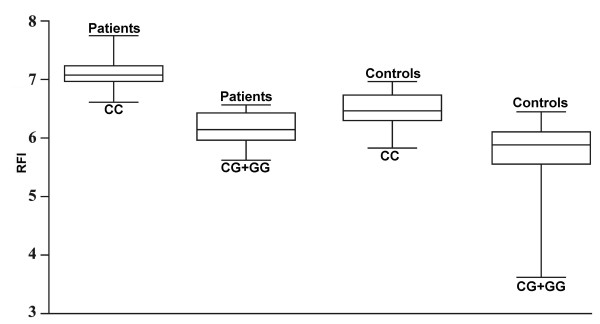
**The intragroup comparison (n = 117 patients, n = 142 controls) of CR1 expression (RFI) with regard to the *CR1 *5507 standard allele homozygotes (CC) versus G mutant allele carriers (CG+GG)**. The data are expressed as whisker box plots; the box represents the 25-75th percentiles, the median is indicated by a bar across the box, the whiskers on each box represent the minimum and maximum level. *P *= 0.0003 when comparing levels between patients and controls.

Distribution of genotypes for *CR1 *C5507G SNP corresponded to the Hardy-Weinberg equilibrium. We found no significant differences in distribution of genotypes, allele and carriage frequencies, between the groups of schizophrenia patients and control subjects (*p *> 0.05, table 2). The intergroup comparisons showed that in the CC genotype the levels of CR1 expression on E, M, and N were higher in patients than in controls (*p *< 0.006), whereas in lymphocytes practically equal levels of CR1 expression in both groups were detected (*p *> 0.05). Further, we compared the levels of CR1 expression on the blood cells of normal allele homozygotes and mutant allele carriers (CG+GG) in patients and controls. Both groups showed increased CR1 expression on E in CC homozygous subjects compared to mutant allele carriers (Figure 2), whereas levels of CR1 expression on L, M, and N did not differ between these genotypes (*p *> 0.05; table 3). No correlation between the *CR1 *5507G carriers and clinical characteristics of patients with schizophrenia as well as smokers and non-smokers in both study groups has been observed. In patients, the fractions of CR1 positive L (CC: 10.45 [5.41] %, CG+GG: 13.51 [4.90] %, *p *= 0.047) and N (CC: 62.95 [10.90] %, CG+GG: 70.75 [14.98] %, *p *= 0.026) were higher in mutant allele carriers, whereas in control subjects the number of CR1 positive E were significantly higher in CC homozygotes (CC: 80.10 [4.54] %, CG+GG: 75.09 [6.48] %, *p *= 0.023).

**Table 2:** Distribution of *CR1 *C5507G genotypes and carriage rates for minor alleles in schizophrenic patients and controls.

		Frequency, absolute (relative)		OR, CI, *p*
		**Patients**	**Controls**	

	CC	75 (0.64)	84(0.59)	

**Genotype**	CG	38 (0.32)	55 (0.39)	

	GG	4 (0.03)	3 (0.02)	*p *= 0.45

**Allele**	C	188 (0.80)	223 (0.79)	

	G	46 (0.20)	61 (0.21)	OR = 1.15, CI: 0.85-1.36, *p *= 0.53

**Carriage**	C	113 (0.98)	139 (0.97)	

	G	42 (0.41)	58 (0.36)	OR = 0.78, CI: 0.66-1.16, *p *= 0.63

The data are presented as absolute numbers with proportions in parenthesis.

**Table 3:** Comparison of CR1 expression on blood cells of schizophrenic patients and healthy controls depending on the CR1 C5507G genotypes.

	CR1 expression in patients			CR1 expression in controls		
**Type of blood cells**	**CC**	**CG+GG**	***p***	**CC**	**CG+GG**	***p***

**Erythrocytes**	7.07 [0.31]	6.15 [0.52]	6.57E-08	6.46 [0.46]	5.87 [0.52]	6.88E-06

**Lymphocytes**	21.70 [8.48]	24.15 [9.33]	> 0.05	19.5 [8.25]	18.50 [8.50]	> 0.05

**Monocytes**	80.80 [49.35]	89.60 [45.88]	> 0.05	48.86 [9.00]	45.45 [16.75]	> 0.05

**Neutrophils**	190.00 [91.30]	174.50 [65.00]	> 0.05	128.00 [42.50]	149.5 [97.00]	> 0.05

### Levels of C1q-CIC and C3d-CIC in the blood

The median level of C1q-CIC in the blood serum was significantly higher in patients compared to controls (patients 27.25 [4.17] ug/mL versus controls: 24.23 [3.88] ug/mL, *p *= 0.017), whereas the levels of C3d-CIC were practically the same in both groups (patients: 14.20 [2.68] ug/mL versus controls14.09 [3.77] ug/mL, *p *= 0.66). No difference in C3d-CIC and C1q-CIC levels was observed between *CR1 *5507 CC homozygotes and mutant allele carriers in patients (*p *> 0.05). In contrast, the G allele carriers in control subjects had significantly elevated C1q-CIC levels (CG+GG: 25.18 [3.16] ug/mL versus CC: 22.27 [3.73] ug/mL, *p *= 0.015). Correlation analysis showed that CR1 expression levels on blood cells in control subjects positively correlated with C3d-CIC levels (rho = 0.44, *p *= 0.049) and negatively correlated with C1q-CIC levels (rho = -0.42, *p *= 0.058). In patients with schizophrenia, no significant correlation between these parameters was observed. Also, CIC levels did not differ between smokers and non-smokers among the patients and controls.

## Discussion

Recent studies provide evidence of the involvement of the complement system alterations in schizophrenia-associated immune system abnormalities including autoimmunity and inflammation [13-17]. In this work, we have extended the current knowledge on the state of the complement system in schizophrenia by studying CR1 expression on blood cells, the levels of CIC bound to CR1 ligands, the products of the complement activation, and the *CR1 *C5507G functional polymorphism in diseased and healthy subjects.

The results obtained for the first time demonstrate an increased expression of CR1 on erythrocytes and subpopulations of leukocytes (L, M, and N), accompanied by the elevated number of CR1 positive E and reduced number of CR1 positive L and M in patients with schizophrenia as compared to healthy subjects. Regarding CIC bound to complement-derived CR1 ligands, the increased levels of C1q-CIC in schizophrenia patients compared to healthy controls were detected, whereas no significant difference in the levels of C3d-CIC between patients and controls was observed.

Changes in CR1 expression on blood cells have been implicated in a variety of diseases (systemic lupus erythematosus, acute immunodeficiency syndrome, rheumatoid arthritis, malarial anaemia, insulin-dependent diabetes, myocardial infarction, etc) associated with immune system dysfunction, development of inflammatory and autoimmune reactions [26,27,29-33]. Depending on the diseased condition, these changes may be determined by either genetic or environmental factors. The present finding of the increased CR1 expression levels on blood cells of schizophrenia patients may be a consequence of the elevated levels of the total population of IC in circulation reported by us previously [34], as well as increased levels of C1q-CIC reported in the present work. As noted in the introduction, binding of erythrocyte CR1 to the complement component C1q attached to IC serves to mediate clearance of IC [5]. In addition, increase of CR1 expression on leukocytes can be induced by tumor necrosis factor-α and interleukin-1 [35,36], which are elevated in the blood of schizophrenic patients [37].

We also observed an enhanced number of CR1 positive E in schizophrenia patients, while fractions of CR1 positive L and M were significantly decreased compared to control subjects. Increased number of CR1 positive E in circulation might be related to the enhanced hematopoiesis in response to cytokines and other inflammatory agents [38], while reduced number of CR1 positive leukocyte subpopulations might be explained by increased rate of apoptosis induced by antipsychotic treatment [39-41].

Distributions of the allele and genotype frequencies of the *CR1 *C5507G SNP in schizophrenia patients do not differ from healthy subjects. Therefore, despite the important role of the complement system in the pathogenesis of schizophrenia [15-22], its type 1 receptor gene C5507G polymorphism does not contribute to the genetic susceptibility to this disease. Further, we compared the levels of CR1 expression on the blood cells of normal allele homozygotes and mutant allele carriers (CG+GG) in patients and controls. Both groups showed increased CR1 expression on E in CC homozygous subjects compared to mutant allele carriers (Figure 2), whereas levels of CR1 expression on L, M, and L did not differ between these genotypes (*p *> 0.05, table 3). Interestingly, Wilson et al. found the association of genomic polymorphism with a cis-acting regulatory element for the expression of CR1 on E [42].

## Conclusions

In conclusion, on the basis of our results we suggested that schizophrenia is associated with the increased expression of CR1 on E, L, M, and N accompanied by the elevated number of CR1 positive E, reduced number of CR1 positive L and M, and increased level of C1q-CIC. Interestingly, the levels of CR1 expression on E were higher in *CR1 *5507 CC homozygotes when compared to those in G allele carriers both among the patients with schizophrenia and control subjects. Further studies in other ethnic groups and populations are needed to confirm our findings.

### Limitations of our study

Some limitations in this study should be noted. Firstly, we analyzed a relatively small sample size (117 schizophrenia patients and 142 healthy subjects). Secondly, all the patients with schizophrenia involved in the present study were receiving antipsychotic treatment.

## Methods

### Study population

In total, 117 patients with paranoid schizophrenia (F20.0) and 142 healthy subjects were enrolled in the study. All subjects were unrelated Armenians living in Armenia. All patients were diagnosed according to the ICD-10 criteria [43] by two independent experienced psychiatrists. The affected subjects (females/males: 60/57, mean age ± SD: 45 ± 10 years, age at the first-onset of illness: mean ± SD: 23 ± 9 years, duration of illness mean ± SD: 19 ± 7 years, smokers/non-smokers: 33/84) were recruited from the clinics of the Psychiatric Medical Center of the Ministry of Health of the Republic of Armenia (MH RA). All patients were receiving typical antipsychotic haloperidol and phenazepamum during their treatment. Healthy volunteers (females/males: 70/72, mean age ± SD: 42 ± 12 years, smokers/non-smokers: 50/72) were recruited from the Erebouni Medical Center MH RA and served as a reference control population. They passed a special examination by two independent experienced psychiatrists proved no personal or family history of mental disorders. All subjects gave their informed consents to provide 10 ml of venous blood for the purposes of this study. The study was approved by the Ethical Committee of the Institute of Molecular Biology of the National Academy of Sciences (NAS) RA.

### Collection of blood samples

Ten mL of morning fasting venous blood was collected from each patient and healthy subject using EDTA as anticoagulant.

### Genomic DNA extraction from the whole blood

Genomic DNA samples were isolated from fresh blood by the standard phenol-chloroform procedure [44] and stored at -30°C until further use.

### Genotyping analysis of CR1 C5507G polymorphism

All DNA samples were genotyped for *CR1 *C5507G polymorphism using polymerase chain reaction with sequence specific primers (PCR-SSP) under the conditions described elsewhere [45]. The primer sequences were:

allele C, forward: 5'ATCCGCTGCACAAGTGACCC;

allele G, forward: 5'ATCCGCTGCACAAGTGACCG;

constant reverse: 5'CCCTACTAAATCTGGACCTCATC.

The presence/absence of allele-specific amplicons was visualized by electrophoresis on 2% agarose gel and ethidium bromide fluorescence in reference to a molecular weight marker.

### Isolation of erythrocytes and blood plasma from the whole blood

Blood samples were centrifuged (700 g × 10 min) at room temperature. After centrifugation plasma was separated and a buffy coat was removed. A pellet of E was washed 3 times by resuspension in BD CellWASH™ solution (BD Biosciences, CA, USA) followed by centrifugation (700 g × 10 min) at 4°C. After washing the E resuspended in the same solution were counted and adjusted to a final concentration of 10^6 ^cells/mL.

### Isolation of leukocytes from the whole blood

Blood samples were treated with BD lysis solution (BD Biosciences, CA, USA) to lyse E, and then centrifuged (700 g × 10 min) at room temperature. A pellet, containing leukocytes, was washed 3 times by resuspension in BD CellWASH™ solution followed by centrifugation (700 g × 10 min) at room temperature. After washing the leukocytes were resuspended in the same solution, counted and adjusted to a final concentration of 10^6 ^cells/mL.

### Measurement of CR1 expression on blood cells

Suspensions of E and L (100 uL of each) were incubated with FITC-labeled anti-CD35 monoclonal antibody (catalog number 555452, BD Pharmingen, USA) for 1.5-2 hours and then fluorescence at 518 nm (excitation 488 nm) was measured on BD FACScan flow cytometer supplied with BD CellQuest™ Pro 5.2 software (BD Bioscience, USA). Histogram overlays and dot plots were produced by WinMDI 2.9 software package. Representative pictures of gated cell populations and histograms are shown on Figure 1. A matching isotype control (mouse IgG1 isotype control FITC, catalog number 11-4714-42, eBioscience, USA) was used to measure non-specific binding of antibodies to studied cells, and this value indicated in each histogram with a filled curve was subtracted from the raw data. E and subpopulations of leukocytes (L, M, and N) were identified by forward versus side scatter parameters, and surface expression of CR1 in each cell population was quantified. An isotype control was used to estimate the fraction of CR1 positive cells (see Figure 1) which was expressed as a percentage of positive cells among the cells of particular population. Surface expression is given in relative fluorescence intensity units (RFI) as median [interquartile range]. Events with RFI exceeding those of isotype control were considered as CR1-positive, and were gated on the histogram as a separate subpopulation. The median RFI was measured to determine CR1 expression in the marked subpopulation. Histogram overlays and dot plots were produced by WinMDI 2.9 software package.

### Measurement of C1q- and C3d-bound CIC

The levels of C3d- and C1q-bound CIC (C3d-CIC and C1q-CIC, respectively) were measured in plasma by enzyme-linked immunosorbent assay (ELISA) using commercially available kits (IMTEC-C3d-CIC and IMTEC-C1q-CIC, Human GmBH, Germany) according to manufacturer's instructions. The level of C3d-CIC reflects total concentration of CIC with bound C3 fragments (C3b, iC3b, and C3dg), which contain the fragment of the C3 alpha polypeptide chain known as the "d" fragment.

### Statistical analysis

The distributions of genotypes for *CR1 *C5507G were checked for correspondence to Hardy-Weinberg (H-W) equilibrium. In order to find potential relevance of the selected *CR1 *SNP to schizophrenia, its allele (gene) and phenotype frequencies (carriage rates) in patients and control subjects were compared. The significance of differences between allele and phenotype frequencies calculated on the base of the number of observed genotypes was determined using Pearson's Chi-square test. The odds ratio (OR), 95% confidence interval (CI), and Pearson's *p*-value were calculated. The Mann-Whitney U test was used for evaluation of intergroup differences in the levels of CR1 expression, C1q-CIC, and C3d-CIC and percentage of CR1 positive cells. Survival analysis was performed to evaluate the possible association between clinical/demographic characteristics of schizophrenia patients and genotypes and expression levels observed.

Group statistics, otherwise indicated, was presented as median [interquartile range]. Spearman's rank correlation coefficient (rho) was calculated to assess relationships between measured parameters. *P*-values less than 0.05 were considered significant. Statistical analysis was performed using "Graphpad Prism" (GraphPad Software Inc., USA) software.

## List of abbreviations

CR1: complement receptor type 1; C1q: q subcomponent of complement component 1; C3b: iC3b and C3dg, fragments of complement component 3; C4b: opsonic fragment of C4; FITC: fluorescein isothiocyanate; RFI: relative fluorescence intensity; PCR-SSP: polymerase chain reaction with sequence specific primers; SNP: single nucleotide polymorphism; ELISA: enzyme-linked immunosorbent assay; E: erythrocyte; L: lymphocyte; M: monocyte; N: neutrophil; CIC: circulating immune complexes; OR: odds ratio; CI: confidence interval; ICD-10: International classification of diseases, 10th edition.

## Competing interests

The authors declare that they have no competing interests.

## Authors' contributions

AA isolated blood cells and drafted the manuscript. RZ performed DNA isolation from blood samples and genotyping analysis. AK measured CIC levels in patients with schizophrenia and healthy subjects. DP performed flow cytometry experiments while RA interpreted the data obtained. FM designed primers for PCR-SSP and did statistical analysis. MP performed genotyping study design and accompanied with AB, who generated the idea of the study, developed the final version of manuscript. All authors read and approved the final manuscript.

## Pre-publication history

The pre-publication history for this paper can be accessed here:

http://www.biomedcentral.com/1472-6890/11/10/prepub
